# Genetic adaptation of Tibetan poplar (*Populus szechuanica* var. *tibetica*) to high altitudes on the Qinghai–Tibetan Plateau

**DOI:** 10.1002/ece3.6508

**Published:** 2020-10-01

**Authors:** Chenfei Zheng, Lizhi Tan, Mengmeng Sang, Meixia Ye, Rongling Wu

**Affiliations:** ^1^ Beijing Advanced Innovation Center for Tree Breeding by Molecular Design Center for Computational Biology College of Biological Sciences and Technology Beijing Forestry University Beijing China; ^2^ Center for Statistical Genetics Pennsylvania State University Hershey PA USA

**Keywords:** adaptation, gene flow, genetic loci, population genetic variation, Tibetan poplar

## Abstract

Plant adaptation to high altitudes has long been a substantial focus of ecological and evolutionary research. However, the genetic mechanisms underlying such adaptation remain poorly understood. Here, we address this issue by sampling, genotyping, and comparing populations of Tibetan poplar, *Populus szechuanica* var. *tibetica*, distributed from low (~2,000 m) to high altitudes (~3,000 m) of Sejila Mountain on the Qinghai–Tibet Plateau. Population structure analyses allow clear classification of two groups according to their altitudinal distributions. However, in contrast to the genetic variation within each population, differences between the two populations only explain a small portion of the total genetic variation (3.64%). We identified asymmetrical gene flow from high‐ to low‐altitude populations. Integrating population genomic and landscape genomic analyses, we detected two hotspot regions, one containing four genes associated with altitudinal variation, and the other containing ten genes associated with response to solar radiation. These genes participate in abiotic stress resistance and regulation of reproductive processes. Our results provide insight into the genetic mechanisms underlying high‐altitude adaptation in Tibetan poplar.

## Graphical abstract

In this article, we address this issue by sampling, genotyping, and comparing populations of Tibetan poplar, *Populus szechuanica* var. *tibetica*, distributed at low (~2,000 m) and high altitudes (~3,000 m) of Sejila Mountain on the Qinghai‐Tibet Plateau. We identified clearly classification of these two populations according to altitudinal distribution, and we also detected low population divergence and robust asymmetry gene flows between them. Finally, we identified a hot spot region under selection and associated with environmental variables through scanning of the whole Tibetan poplar genome.

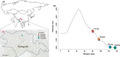

## 1 INTRODUCTION

One of the major goals of evolutionary genetics is to discover the driving forces behind adaptive evolution and their roles in shaping patterns of polymorphism and divergence within and among species (Lin et al., 2018; Savolainen, Lascoux, & Merilä, 2013). Two bottom‐up approaches, population genetics and landscape genomics, are commonly used to identify genes underlying local adaptation. The field of population genetics aims to identify the evolutionary forces, such as natural selection, gene flow, and demographic fluctuations that play dominant roles in driving plant adaptation to local environments. Today, the increasing availability of genome‐wide data is transforming population genetics into population genomics and simultaneously revolutionizing our understanding of local adaptation (Luikart, England, Tallmon, Jordan, & Tab erlet, 2003; Weigel & Nordborg, 2015). Using genome‐wide data analyses, it is possible to elucidate the relative contributions of various evolutionary forces to the current extent and pattern of genetic variation, as well as their potential roles in local adaptation (Cutter & Payseur, 2013; Olson‐Manning, Wagner, & Mitchell‐Olds, 2012; Sella, Petrov, Przeworski, & Andolfatto, 2009). However, using a population genetics or genomics approach has several inevitable drawbacks. For example, it is difficult to detect rare alleles involved in local adaptation, particularly in a complicated demographic context such as during robust gene flow events (Kawecki & Ebert, 2004).

In recent years, landscape genomics has emerged as a valuable alternative approach for identifying adaptive loci that drive local adaptation (Holderegger, Buehler, Gugerli, & Manel, 2010; Manel & Holderegger, 2013; Manel et al., 2010; Sork et al., 2013). The rise of landscape genomics has been expedited by next‐generation sequencing, the increasing availability of public datasets of environmental factors, and the rapid development of computational power (Balkenhol et al., 2019; Rellstab, Gugerli, Eckert, Hancock, & Holderegger, 2015). For example, high‐throughput sequencing technology allows the quantification of numerous allele variants across the whole genomes of many individuals (Andrews, Good, Miller, Luikart, & Hohenlohe, 2016; Luikart et al., 2003). Likewise, environmental data can be obtained at high resolution using accurate remote sensing devices (Anderson & Gaston, 2013; Pettorelli et al., 2005). Increased computational power enables analyses of the large datasets, thus, generated in a reasonable amount of time (Kidd & Ritchie, 2006; Paul & Song, 2012). Unlike traditional approaches to testing outlier loci, landscape genomics has the potential to discern adaptive patterns by identifying genetic variants coupled with particular environmental factors. Recently, numerous studies of local adaptation combining both population genetic and landscape genomic approaches have been reported in various species, notably in forest trees such as *Pinus*, *Picea*, and *Populus* (Eckert et al., 2010; Geraldes et al., 2013, 2014; Grivet et al., 2013; Keller, Levsen, Ingvarsson, Olson, & Tiffin, 2011).

Various types of forest trees are serving as model species that can provide information about demographics and adaptive processes in forest ecosystems through population genomics or landscape genomics (Sork et al., 2013). *Populus* is a globally distributed tree genus that is native to the Northern Hemisphere and contains nearly 30 species. Poplars are pioneer species and ecologically important trees in their habitats. Due to several advantages of poplar species, including rapid growth, relatively small genome size (<500 Mbp), suitability for efficient genetic transformation, and ease of propagation in tissue culture, they have become important model organisms for studies of forest tree species with well‐developed genetic and genomic resources (Street & Tsai, 2010; Wullschleger, Weston, DiFazio, & Tuskan, 2013). Several studies of population genomics and landscape genomics of local adaptation have been reported in *Populus trichocarpa* (Evans et al., 2014; Geraldes et al., 2014; Holliday, Zhou, Bawa, Zhang, & Oubida, 2016; Porth et al., 2015; Zhou, Bawa, & Holliday, 2014), *Populus balsamifera* (Fitzpatrick & Keller, 2015; Keller et al., 2011; Keller, Levsen, Olson, & Tiffin, 2012), *Populus* *alba* (Stölting et al., 2015), *Populus* *tremula* and *Populus* *tremuloides* (Wang, Street, Scofield, & Ingvarsson, 2016a, 2016b), and *Populus* *deltoids* (Fahrenkrog, Neves, Resende, Dervinis, et al., 2017; Fahrenkrog, Neves, Resende, Vazquez, et al., 2017). However, very limited information is available about high‐altitude adaptation in ecologically and economically important endemic alpine species such as Tibetan poplar *Populus szechuanica* var. *tibetica*, which is distributed on the Qinghai–Tibetan Plateau (QTP).

The Tibetan poplar is a perennial woody plant belonging to *Populus* sect. *Tacamahaca* that is endemic to the QTP and mainly distributed in Sichuan and Tibet along an altitude gradient from 2,000 to 4,500 m (Shen, 2014). Recent studies have concentrated mostly on the genetic diversity, phenotypic, and physiological mechanisms accounting for its adaptation to harsh environmental conditions including low temperature, strong solar radiation, and poor soil (Shen et al., 2014; Tang, Pubu, & Cidan, 2012). However, the genetic mechanisms underlying local adaptation to increasing altitudes in Tibetan poplar remain unclear. Here, we investigated the genetic diversity and genetic adaptations of Tibetan poplar at low (~2,000 m) to high altitudes (~3,000 m) to investigate its genetic adaptation to this harsh high‐altitude environment using genome‐wide single‐nucleotide polymorphism (SNP) data obtained from genomic resequencing technologies.

## 2 MATERIALS AND METHODS

### 2.1 Sampling strategy and DNA extraction

**FIGURE 1:**
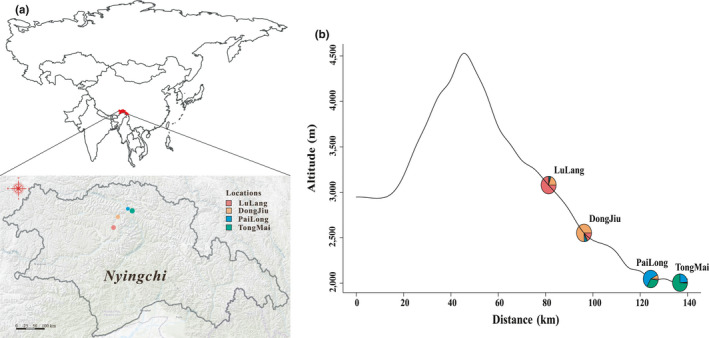
Sampling locations of *Populus szechuanica* var. *tibetica* on Sejila Mountain. All 400 trees analyzed were collected from four sampling sites (a) along an altitudinal gradient (b). The area of each circle represents the sample size. Admixture proportion was assigned based on both original sample sites and a Q‐matrix generated by Admixture tools with *K* = 4

A total of 400 samples were collected from Sejila Mountain in southeastern Tibet along an altitudinal gradient of 2,000–3,000 m in the summers of 2013 and 2014 (Figure 1; Table S1). These samples were clustered into two altitude groups: high (LL and DJ) and low (PL and TM). However, they were distributed continuously throughout the study area. Each individual was collected at least 30 m from others to prevent the selection of clones. Cuttings of each sample were planted in cultivation medium composed of vermiculite and perlite in a greenhouse at Beijing Forestry University. Approximately 0.2 g newly emerged leaves was prepared from the cuttings for DNA extraction to prevent insect DNA contaminants. Total genomic DNA was extracted using the DNAsecure Plant kit (Tiangen Biotech (Beijing) Co., Ltd.) following the protocol. After quality control of extracted DNA using 1% agarose gel electrophoresis and ultraviolet spectrophotometry, at least 1.5 µg DNA from each sample was prepared for genome‐wide resequencing.

### 2.2 SNP calling and data filtering

Genomic DNA libraries of 500‐bp fragment size were constructed and then paired‐end sequenced on the Illumina sequencing platform (Hiseq 2000) at the Novogene Bioinformatics Institute (Beijing, China) in 2015. Raw reads were trimmed to remove (a) adapter sequences, (b) raw reads containing more than 15 N bases (10% of 150 nt) in a single read, and (c) raw reads containing more than 75 nt low‐quality bases (*Q* ≤ 5). Trimmed reads were mapped to the *P. trichocarpa* genome version 3.0 (https://genome.jgi.doe.gov/) using BWA (mem –t 4 –k 32 ‐M; Li & Durbin, 2009) and SAMtools program “rmdup” to remove duplications (Li et al., 2009). Only reads with at least an 85% match to the reference genome were retained for subsequent SNP calling using the SAMtools program “mpileup” with the following parameters: ‐E –C 50 –DS –m 2 –F 0.000911 –d 50,000 (Li et al., 2009). Next, we removed SNPs with minor allele frequency (MAF) ≤ 0.1, missing genotype rate < 20%, minimum depth 10×, and maximum depth 20× using VCFTools (Danecek et al., 2011). Ultimately, 490,363 SNPs were maintained for subsequent analyses. Features of these SNPs were annotated using SnpEff software (ver 4.0; Cingolani et al., 2012) against *P. trichocarpa* genome v. 3.0.

Pairwise kinship among 400 samples was inferred using the program King 2.2.3 with all filtered SNPs (Manichaikul et al., 2010). All duplicate individuals were removed, and 348 independent individuals were retained for subsequent analyses (Figure S1).

### 2.3 Population structure and divergence

One of the major assumptions employed in inferring population structure was that there were no spurious correlations among the measured variables. Therefore, the physical and linkage disequilibrium‐correlated SNPs needed to be pruned before population structure estimation. First, SNPs were thinned by randomly selecting one SNP from a 10‐Kbp window size, leaving 31,793 SNPs retained for the subsequent population structure and divergence estimation.

**FIGURE 2:**
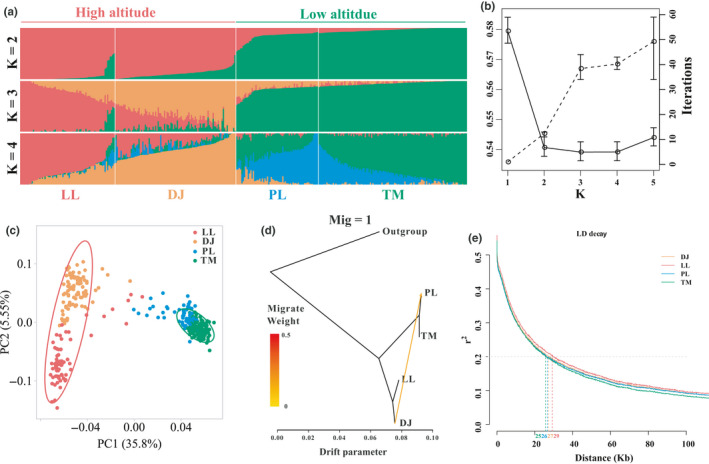
Population structure of *Populus szechuanica* var. *tibetica*. (a) Admixture of 348 unrelated samples illustrated for *K* = 2, 3, 4. (b) CV error (solid lines) and number of iterations (dashed lines) for each K. Average value and standard error were calculated by dataset (from 5‐ to 25‐Kbp window size). (c) PCA of population structure. Confidence intervals are marked with ellipses, red for the high‐altitude group (LL & DJ), and green for the low‐altitude group (PL & TM). (d) Gene flow events inferred by TREEMIX software. (e) Pattern of LD decay for all sample sites using *r* ^2^ values. The number indicates the physical distance beyond which the *r* ^2^ value was below .2 for each locality

We used both model‐independent and model‐dependent methods to infer population structure from resequencing data. Model‐independent principal component analyses (PCAs) were performed using the package GCTA (Yang, Lee, Goddard, & Visscher, 2011). A more precise population genetic structure was inferred using Admixture software (Alexander, Novembre, & Lange, 2009). The predefined genetic cluster value (*K*) was set from 1 to 5. The number of iterations for convergence for each *K* is given in Figure 2b. We then selected the most probable number of subpopulations according to the maximum marginal likelihood value based on the minimum cross‐validation (CV) errors (Figure 2b). Because there was a subtle difference in the CV error when *K* = 3 versus *K* = 4, we also pruned the dataset by randomly selecting one SNP each from 5‐, 15‐, 20‐, and 25‐Kbp window sizes and repeating the population structure analysis. Individuals collected from one sample site but clustering with other sites based on the Q‐value measured by Admixture with *K* = 4 were treated as admixtures (Figure 1b).

We also performed analyses of molecular variation (AMOVAs) using Arlequin version 3.5 (Excoffier & Lischer, 2010) to assess the distribution of total genetic variation. For the AMOVA, we combined the four sample sites into two altitudinal groups (high: LL & DJ; low: PL & TM).

### 2.4 Gene flow and migration events

According to the continual distribution of Tibetan poplar on Sejila Mountain and lack of a geological barriers separating our four sample sites, gene flow might be a driving force for the population structure and genetic diversity. Therefore, we inferred the population divergence and gene flow by reconstructing the maximum likelihood phylogenetic tree based on allele frequency data using Treemix software version 1.12 (Pickrell & Pritchard, 2012). Three individuals of *P. trichocarpa* (downloaded from the Joint Genome Institute database http://phytozome.jgi.doe.gov) were used as the outgroup. The migration event parameter was set from 1 to 4. Pruned SNPs (*n* = 31,793) were used to estimate migration events.

### 2.5 Estimation of genetic parameters

After inferring population structure and gene flow events, we estimated several genetic parameters, as detailed below. All of the filtered SNPs (*n* = 490,363) were used for calculating these genetic parameters.

1. Population fixation index (*F*_ST_). The population fixation index (*F*_ST_) was calculated for all six pairwise comparisons of four sample sites using a sliding window method implemented in VCFTools with a window size of 80 Kbp and a step size of 10 Kbp. A multiple regression on matrices (MRM) test was performed using the package *ecodist* implemented in R software (https://www.r‐project.org/) with 1,000 permutations to test whether genetic distance was correlated with geological distance for the four sample sites.
2. Nucleotide diversity estimation (*π*). Sliding window estimates of nucleotide diversity (*π*) were calculated for all four sample sites with the same parameters used in the *F*_ST_ calculation. We also compared the nucleotide diversity of different genomic regions, such as intergenic, exon, intron, UTR, upstream, and downstream regions.
3. Linkage disequilibrium (LD). The squared correlation coefficient (*r* ^2^) was calculated for LD of SNPs in a 100‐Kb window for all four sample sites using Plink software (‐‐ld‐window‐kb 100). The general pattern of LD decay was then estimated using the software LD decay with default parameters (Zhang, Dong, Xu, He, & Yang, 2019). A plot of the LD decay rate against physical distance was generated using R software (https://www.r‐project.org/).

### 2.6 Signatures of divergent selection

To investigate the mechanisms underlying adaptation to altitudinal gradients, whole‐genome scanning (490,363 SNPs) was performed with a *F*_ST_ outlier approach to ascertain selective signals, executed in BayeScan software v2.1 (Foll & Gaggiotti, 2008). In this method, the *F*_ST_ coefficients are separated into two components, a locus‐specific component (*α*) and a population‐specific component (*β*). Selective signatures can be detected when *α* is extremely different from zero. The minimum false discovery rate at which the locus may be under significant selection was calculated as a *q*‐value. Prior odds of the selection model were set at 10,000 to reduce false‐positive results under a variety of demographic events (Lotterhos & Whitlock, 2014). Loci identified based on the BayeScan method (*q*‐values < 0.01) were considered as putative SNPs under selection (selSNPs). These selSNPs were retained for the subsequent environmental association analysis.

### 2.7 Environmental association analysis

Seven altitudinal climatic variables were examined as possible factors explaining loci under selection: solar radiation (srad), precipitation (prec), wind speed (wind), water vapor pressure (vapr), average temperature (tavg), minimum temperature (tmin), and maximum temperature (tmax). All climatic variables were acquired from the WorldClim global climate database (http://worldclim.org) version 2.0 (Fick & Hijmans, 2017) implemented in ArcGIS 10.6 (http://desktop.arcgis.com), corresponding to recent historical conditions (1970–2000) with a spatial resolution of 2.5 arc‐min. Since there was a high degree of correlation among environmental factors, we chose only alt, srad, and tavg as independent factors in environmental association analyses. The yearly standard average value of each climate variable was used.

Here, we applied a Bayesian linear mixed‐based model in Bayenv2 (Günther & Coop, 2013). For a given genetic variant, Bayenv2 tests whether a model with environmental factors included is more suitable than the null model, from which environmental factors are excluded. A variance–covariance matrix was constructed by running a Markov chain Monte Carlo algorithm for 10,000 iterations based on allele frequencies accounting for the population structure using the pruned SNPs dataset generated by a 10‐Kbp random window (*n* = 31,793). The variance–covariance matrix was then used to control for evolutionary history during the process of BF calculation using Bayenv2. BF was calculated using normal environmental correlation analysis for each SNP. Additionally, a nonparametric test, which excluded the covariance structure among populations, was performed for calculation of the nonparametric Spearman's rank correlation coefficient *ρ* using the parameter ‐c. Those loci that ranked in the top 1% of BF and the absolute value of *ρ* were identified as putative loci associated with a certain environmental factor. Due to the limitation imposed by the small number of sample sites in this study (*n* = 4), we further performed a permutation test by reassigning individuals to sample sites and repeating the Bayenv2 analysis 1,000 times. The possibility that the recalculated values of BF and absolute *ρ* were greater than the original values was defined as the *p*‐value. Putative environment‐associated SNPs (eaSNPs) would be identified as such under a threshold (*p*‐value < .01).

### 2.8 Candidate gene annotation

The gene IDs of potential selected genes located in selective regions were extracted from the latest general feature format (GFF) file of the *P. trichocarpa* genome (RefSeq assembly accession: GCF_000002775.4) using a custom Python script. We then converted gene IDs to gene ontology (GO) IDs using the *bitr* function and the *toTable* function to extract GO terms in the clusterProfiler package (Yu, Wang, Han, He, 2012) in R. The annotation database used in this study was acquired using the *AnnotationHub* package (Morgan, Carlson, Tenenbaum, & Arora, 2019). The accession number of the *P. trichocarpa* database is “AH66282.” The GO annotation diagram was plotted using a custom script implemented in R software.

## 3 RESULTS

### 3.1 Resequencing data SNP calling

A total of 15.6 terabases (Tb) were mapped onto 394 Mb, providing approximately 88% (84.53%–91.78%) coverage of the *P. trichocarpa* genome, with an average 15 × (10.08×–19.7×) depth of sequencing. A total of 490,363 SNPs were retained for subsequent analyses after SNP filtering. Among those SNPs, 39,428 were out of Hardy–Weinberg equilibrium (HWE) under a threshold *p*‐value (10^–4^). Approximately 48.54% of 490,363 SNPs were located in intergenic regions, and 12.88% were detected in exon regions (Table S3).

### 3.2 Population structure

We retained 31,793 unrelated SNPs for sequential population structure inference. In PCA, all 348 unrelated samples from the four sampling locations clustered into two spatially separated groups associated with different altitudes (Figure 2c). The first component could explain 35.8% of the differences among variables. A more comprehensive assessment of the stratification present in Tibetan poplar was obtained using Admixture software (Alexander et al., 2009). First, we inferred the probable population structure by setting subpopulation numbers from 1 to 5 and chose the most suitable *K* value by selecting the minimum CV error using 31,793 SNPs pruned by 10‐Kbp random windows (Figure 2a,b). The most suitable value for *K* was 3, which had the minimum CV error. However, the difference between the CV error when *K* = 3 and *K* = 4 was very slight. Then, we compared the differences among the CV errors of different pruned SNP datasets generated from 5‐, 15‐, 20‐, and 25‐Kbp windows (Table S4). There was no significant divergence between these values when *K* = 3 and *K* = 4 (Figure 2b). Given the population structure and the number of sample sites, we chose *K* = 4 as the most suitable value for population structure in the subsequent analysis.

### 3.3 Migration event inference

A common migration event indicating gene flow in the direction from high‐altitude (DJ) to low‐altitude (PL) sites was detected in all inferred trees. (Figure 2d & Figure S2). Another gene flow event, which was conditional on three or four migration events, was detected from the other high‐altitude (LL) to low‐altitude (TM) sites (Figure S2). Without exception, the direction of these migration events was from high to low altitude.

### 3.4 Genetic parameters

The average *F*_ST_ value of the four sampling sites was 0.050, ranging from 0.006 (PL against TM) to 0.083 (LL against TM), indicating that there was little genetic differentiation among the four sampling locations (Table S5). Almost 96% of genetic variation was attributable to variation within sample sites, whereas only 3.64% of the variation was attributable to differences between the two altitude groups (Table 1).

**TABLE 1:** Analysis of molecular variance (AMOVA) among *Populus szechuanica* var. *tibetica* along an altitudinal gradient

Source of variation	*df*	Sum of squares	Variance component	Percentage of variation
Among groups	1	75,067.180	191.75251	3.64
Among sample sites within groups	2	16,206.346	18.46357	0.35
Within sample sites	692	3,495,412.873	5,051.17467	96.00
Total	695	3,586,686.399	5,261.39075	100

The average nucleotide diversity (*π*) was 7.26 × 10^–5^. The maximum *π* value was detected at sample site PL (7.57 × 10^–5^), and the minimum was detected at sample site LL (6.95 × 10^–5^). The four sample sites exhibited significant differences in nucleotide diversity (ANOVA, *p* < 2 × 10^–16^). In addition, the average *π* of the intergenic region was 2.2 × 10^–4^, which was significantly greater than that of the genic region (*π* = 4.37 × 10^–5^). The results of ANOVA indicated that *π* values significantly differed among divergent genetic regions (Figure S3).

The average distance at which LD values decayed below 0.2 was almost 26 Kbp. The rate of LD decay for the low‐altitude group was much quicker (~25.5 Kbp) than that for the high‐altitude group (~28 Kbp; Figure 2e). The slowest LD decay rate was detected at LL (~29 Kbp), and the fastest at TM (~25 Kbp; Figure 2e; Figure S4).

### 3.5 Signature of natural selection

A total of 7,064 SNPs under selection (selSNPs) were identified based on the *F*_ST_ outlier approach implemented in BayeScan software (*q*‐value < 0.01). Approximately 62.8% of outliers (*n* = 4,441) were out of HWE (*p* = 1 × 10^–5^), and these loci were located in 531 unique genetic regions. The average population index (*F*_ST_) of these selSNPs was 0.181 (range: 0.150–0.271). The locus‐specific component (*α*) ranged from 1.116 to 2.072, indicating that these outlier loci were undergoing continuous directional (divergent) selection. The highest *F*_ST_ (0.271) was harbored in gene LOC18098853, which encodes a disease resistance protein and is homologous with At4g27220 in *Arabidopsis*. This gene is involved in resistance to *Verticillium wilt* in *Arabidopsis* (Li et al., 2018).

### 3.6 Environmental association analyses

**FIGURE 3:**
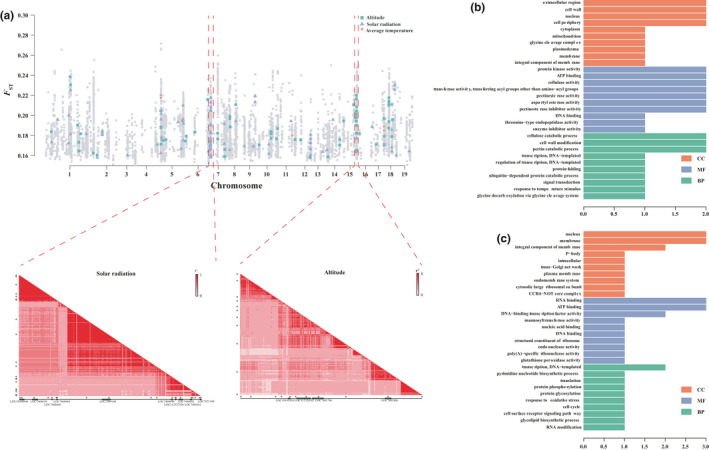
Putative genomic regions under natural selection. (a) Natural selection signals estimated using the BayeScan method (above) and the LD block pattern for SNPs located in the hotspot selective region (below). Gray spots represent the putative selective SNPs identified by BayeScan. Colored spots represent eaSNPs associated with different environmental factors. (b) GO plot for genes located in the hotspot region associated with altitudinal gradient. (c) GO plot for solar radiation‐associated genes located in the hotspot region on chromosome 6

A set of 74 unique selSNPs were associated with altitudinal gradients (altSNPs) based on the hierarchical distribution of BF and absolute *ρ*. Most of these (*n* = 69) were retained after permutation tests. Similarly, 82 selSNPs were associated with variation in solar radiation (sradSNPs) under the criteria of permutation (*p* < .01). However, only 6 selSNPs were associated with average temperature (tavgSNPs; Figure 3a). A common SNP (Chr16: 1,698,907), located in gene LOC112324539, was associated with both altitude and solar radiation. This gene encoded a putative receptor‐like protein kinase which was orthologous with At3g47110 in *Arabidopsis*. Unfortunately, we did not identify any common SNPs that were associated with all three environmental factors (Figure S5a).

One hotspot region, located on chromosome 6 from 26.21 to 26.88 Mbp, exhibited robust signals associated with solar radiation (Figure 3a). This region housed 20 sradSNPs, and 55% of which (11 of 20) were harbored in 10 genes (Figure 3a). The average LD value (*r* ^2^) for 284 SNPs from this region was 0.501, forming three main LD block regions (Figure 3a; Table 2). Another hotspot region, located on chromosome 15 from 13.39 to 14.00 Mbp, contained 11 SNPs associated with altitudinal gradients; four of these were located in four unique genes (Figure 3a).

**TABLE 2:** Candidate environmental factor‐associated genes detected in hotspot selective regions

GeneID	CHR	STR	END	Description	GOID	GO Term	Environment factor
18100694	6	26214002	26218369	Protein PHOX1			Srad
7468658	6	26222031	26224755	MADS‐box transcription factor 47			Srad
7468660	6	26248280	26252031	Hydroxyproline O‐arabinosyltransferase 1			Srad
7468669	6	26349371	26353166	Probable receptor‐like protein kinase At1g80640	GO:0005886 GO:0005524 GO:0004675 GO:0007166 GO:0006468	Plasma membrane ATP binding Serine/threonine kinase activity Cell surface receptor signaling pathway Protein phosphorylation	Srad
7489148	6	26603544	26606487	Stromal cell‐derived factor 2‐like protein	GO:0016020 GO:0000030 GO:0006486	Membrane Mannosyltransferase activity Protein glycosylation	Srad
7468690	6	26748943	26753022	Transmembrane 9 superfamily member 8	GO:0016021	Integral component of membrane	Srad
112327926	6	26758471	26762881	Uncharacterized LOC112327926			Srad
7468692	6	26768302	26775056	Probable protein phosphatase 2C 60			Srad
7489161	6	26807445	26814907	Protein RRC1			Srad
7471349	6	26885203	26890145	Uncharacterized LOC7471349			Srad
18105959	15	13543391	13546165	Squalene monooxygenase			Alt
112324382	15	13566621	13571204	Uncharacterized LOC112324382			Alt
7481796	15	13574767	13581291	Uncharacterized LOC7481796			Alt
7481806	15	13858532	13863900	Coatomer subunit delta			Alt

### 3.7 Annotation of environment‐associated genes

In total, 58 unique genes contained eaSNPs associated with at least one environmental factor (Table S6). One gene (LOC18098834) encoded branched‐chain‐amino‐acid aminotransferase‐like protein 1, associated with both altitude and average temperature. Another gene (LOC112324539) was associated with altitude and solar radiation (Table S6 and Figure S5b). Gene ontology (GO) analysis indicated that these unique genes belonged to 48 GO terms (Figure S5c). The maximum number of GO terms was 3 (ATP binding), in the molecular function (MF) category.

A total of 68 genes were located in a hotspot on chromosome 15, which was associated with responses to altitudinal variation. The GO term analysis indicated that these genes were mainly involved in processes such as manufacture of cell wall components (GO:0005618; GO:0042545), catabolic processes (GO:0006511; GO:0030245; GO:0045490), and signal transduction (GO:0007165; Figure 3b). Most of the altSNPs (7 of 11) were located in intergenic regions, but four altSNPs were harbored in four unique genes (Table 2).

Similarly, 80 genes located in a hotspot region on chromosome 6 were associated with responses to variations in solar radiation. The GO term analysis indicated that these genes were involved in several processes, including manufacture of cell membrane components (GO:0005886; GO:0016020; GO:0016021) and modification of proteins (GO:0006486; GO:0006468; Figure 3c). One gene (LOC7468695) encoded phospholipid hydroperoxide glutathione peroxidase 1 protein, which is involved in the response to oxidative stress (GO: 0006979). This gene contained two SNPs under selection, identified by the BayeScan method. Although Bayenv2 detected no direct signal that these two selSNPs were associated with solar radiation, the robust LD (0.906, range: 0.875–0.94) between this gene and nearby sradSNPs indicated that this gene may play a role in responding to variations in solar radiation.

## 4 DISCUSSION

### 4.1 Asymmetric gene flow in the downhill direction

Several gene flow events from high to low altitude were detected in this Tibetan poplar population. Given the relatively long duration of flowering and long‐distance pollen and seed dispersal of *Populus* species (Ingvarsson, 2010; Vanden Broeck et al., 2004), high gene flow and introgression events have been documented among populations of a single species and multiple related species (Chhatre, Evans, DiFazio, & Keller, 2018; Fahrenkrog, Neves, Resende, Dervinis, et al., 2017; Ma et al., 2018). The Tibetan poplar populations on Sejila Mountain have an overlapping flowering period that runs from late April to mid‐May in the low‐altitude group and throughout May in the high‐altitude group. Furthermore, the sample sites of Tibetan poplar in this study were connected by a river. The combination of overlapping flowering period and shared habitat characteristics enables pollen‐driven gene flow within the entire Sejila population, leading to the potential combination of gene pools and reduction of genetic variation among populations. Wind power might be the driving force for gene flow in the downhill direction observed in this study (Figure S6). The nearer sample sites are to each other, the greater is the potential for gene flow between them (Sharma & Khanduri, 2007). The apparent absence of gene flow between TM and PL (~20 km) might result from a slight divergence of wind power. All analyses detected common gene flow events from DJ to PL, whereas the longest distance (~70 km) gene flow occurred was from LL to TM (Figure S4). Such long‐distance gene flow mediated by either pollen or seeds has been documented for a great diversity of tree species, as reviewed by Kremer et al. (2012).

### 4.2 Genetic adaptation to an altitude gradient

Two hotspot regions were detected, one involved with responses to increasing altitude and the other with solar radiation. Four genes were associated with altitudinal gradients, and 10 were associated with solar radiation. One gene was orthologous to At3g47110, a leucine‐rich repeat receptor‐like kinase gene found in *Arabidopsis thaliana*. This LRR protein interacted with a FERRIC REDUCTASE DEFECTIVE3 (FRD3), which is involved in citrate efflux transportation and sustained microspore development during pollen tube growth in *A. thaliana* (Muschietti & Wengier, 2018). Another gene encoded MADS‐box transcription factor 47, which is associated with solar radiation. The MADS‐box transcription factors participate in floral organ initiation and identity, partially through negative regulation of brassinosteroid (BR) signal transduction (Duan et al., 2006). Brassinosteroids participate in the regulation of multiple biological processes, including abiotic stress resistance (Bartwal, Mall, Lohani, Guru, & Arora, 2013; Takahashi & Shinozaki, 2019) and developmental processes including flowering time, male fertility, pollen development, and woody formation (Clouse, 2011; Du et al., 2020; Gruszka, 2013; Ye et al., 2010).

Other genes located in these hotspot selective regions may also play roles in abiotic stress resistance and flowering time. For instance, the phospholipid hydroperoxide glutathione peroxidase 1 proteins (GPXs) are a group of proteins that protect cells from oxidative damage generated by a reactive oxygen species (ROS; Rodriguez Milla, Maurer, Huete, & Gustafson, 2003). Five GPX genes have been identified in the desert poplar *Populus euphratica* (Meng & Wu, 2017). Strong signals of balancing selection/local adaptation were detected in the *peGPX1* gene: an excess of mid‐frequency alleles, high intraspecific nucleotide diversity (distributed in the upper tail of the simulated neutral model), and extensive LD. However, no selective signatures for other *peGPX* genes have been identified in the desert poplar. GPX also reportedly plays complex roles in diverse developmental processes. In *Arabidopsis*, extremely high expression levels of *AtGPX1* were detected in leaf cell cultures, in mesophyll protoplast cultures, and in shoot apical meristems, indicating its physiological importance during shoot development (Bela et al., 2015). Based on this evidence, we hypothesize that Tibetan poplar adapted to higher altitudes partially through sustaining functions related to reproduction under abiotic stress, though more details about how these genes regulate high‐altitude adaptations in Tibetan poplar remains to be elucidated.

The QTP has been undergoing warming since the 1950s (Kuang & Jiao, 2016). Moreover, the annual precipitation in its eastern and southeastern parts, near our sample sites, is decreasing (Kuang & Jiao, 2016). Thus, the Tibetan poplar of Sejila Mountain will face challenges related to climate change in the near future. Compared to individuals habituated to low‐altitude sites, those at high altitudes will be better able to adapt to climate change. However, individuals at low altitudes could gain beneficial alleles through downhill gene flow in the high‐altitude trees. The interaction between gene flow and natural selection will drive adaptation to cope with environmental changes in the entire Tibetan poplar population in the southeastern QTP.

The effect of using small sample sites on landscape genomic analysis cannot be ignored. Briefly, at larger sample sites, a greater number of individuals are sampled per site, and a greater number of genome‐wide SNPs increase the power of genomic analysis for detecting loci under selection in natural populations, especially for perennial woody plants (Sork et al., 2013). However, in practice, due to the limitation of resources, a balance must be struck between the total number of samples from each site and the total number of localities that can be sampled. If more individuals are sampled from each locality, a more accurate estimation of allele frequency can be generated, improving the power of analysis based on estimates of genetic differentiation among populations. However, increasing the number of sampling sites enables more robust detection of natural selection, as more sampling locations can better represent a range of environmental variable values across the study area (De Mita et al., 2013). In this study, the genome‐wide SNPs and the large number of samples provide insight into how natural selection has shaped Tibetan poplar over an altitudinal gradient. However, the use of only four sample sites, all within a relatively narrow altitudinal gradient, limits our ability to draw conclusions about the genetic response to environmental variables. This shortcoming should be addressed in further studies.

## 5 CONCLUSION

This is the first attempt to elucidate the intricate genetic structures associated with high‐altitude climate adaptation in the QTP endemic woody plant Tibetan poplar, *Populus szechuanica* var. *tibetica*. Integrating population genomic (BayeScan) and landscape genomic (Bayenv2) methods, we detected two hotspot regions with robust signals of natural selection associated with altitude and solar radiation. These regions comprised 14 genes that are mainly involved in abiotic stress resistance and sustaining successful reproduction. Therefore, we hypothesize that Tibetan poplar adapted to high altitude partially through sustaining successful reproduction under conditions of environmental stress. The interaction between gene flow and natural selection drives local adaptation in this population of Tibetan poplar. This paper will be useful for understanding how various evolutionary forces, including natural selection and environmental factors, drive local adaptation to altitudinal differences in Tibetan poplar.

## CONFLICT OF INTEREST

The authors state that there is no conflict of interest.

## AUTHOR CONTRIBUTIONS

**Chenfei Zheng:** Formal analysis (equal); resources (lead); writing – original draft (equal); writing – review and editing (equal). **Lizhi Tan:** Formal analysis (supporting); software (supporting). **Mengmeng Sang:** Formal analysis (supporting); software (supporting). **Meixia Ye:** Formal analysis (equal); software (equal). **Rongling Wu:** Conceptualization (lead); funding acquisition (lead); supervision (lead); writing – original draft (equal); writing – review and editing (lead).

## Supporting information

Appendix S1

## Data Availability

• All SNP data: https://datadryad.org/review?doi=doi:10.5061/dryad.5tk1mc7. • Pruned SNPs: https://datadryad.org/review?doi=doi:10.5061/dryad.5tk1mc7. • Climate data: https://datadryad.org/review?doi=doi:10.5061/dryad.5tk1mc7.
