# Supplementary material for: Genetic adaptation of Tibetan poplar (Populus szechuanica var. tibetica) to high altitudes on the Qinghai–Tibetan Plateau
Source: Ecol Evol. 2020 Oct 1;10(20):10974–85. doi: 10.1002/ece3.6508 (PMC7593140; doi:10.1002/ece3.6508)
Supplement: Supplementary file 1 — Appendix S1 [file ECE3-10-10974-s001.pdf]

Supplemental Information for:

Genetic adaptation of Tibetan poplar (*Populus szechuanica var. tibetica*) to high altitudes on the Qinghai-Tibetan Plateau

Chenfei Zheng[1], Lizhi Tan[1], Mengmeng Sang[1], Meixia Ye[1], *Rongling Wu[1,2]

[1]Center for Computational Biology, College of Biological Sciences and Technology, Beijing Forestry University, Beijing 100083, China

[2]Center for Statistical Genetics, Pennsylvania State University, Hershey, PA 17033, USA

[*]Corresponding author: rwu@bjfu.edu.cn or rwu@phs.psu.edu

Supplementary Table 1 Location for sampling of *Populus szechuanica var. tibetica* populations in Sejila Moutain

Pop Code	Location	Average Altitude (Range)	Latitude (N)	Longitude (E)	Sample Size
LL	Lulang	2984 (2770~3191) m	94°72	29°75	107
DJ	Dongjiu	2590 (2359~2789) m	94°82	29°96	94
PL	Pailong	2042 (1834~2270) m	95°05	30°12	83
TM	Tongmai	2026 (1764~2277) m	95°15	30°08	116

Supplementary Table 2. Correlation among 7 environment variables and altitude

	srad	prec	wind	vapr	tavg	tmax	tmin	alt
srad	1	-0.720	0.414	-0.558	-0.536	-0.557	-0.511	0.549
prec	-0.720	1	-0.926	0.976	0.969	0.976	0.960	-0.972
wind	0.414	-0.926	1	-0.985	-0.990	-0.985	-0.993	0.988
vapr	-0.558	0.976	-0.985	1	0.999	0.999	0.997	-0.999
tavg	-0.536	0.969	-0.990	0.999	1	0.999	0.999	-0.999
tmax	-0.557	0.976	-0.985	0.999	0.999	1	0.996	-0.999
tmin	-0.511	0.960	-0.993	0.997	0.999	0.996	1	-0.998
alt	0.549	-0.972	0.988	-0.999	-0.999	-0.999	-0.998	1

Supplementary Table 3 Annotated features for single nucleotide polymorphism (SNP) variants in *P. szechuanica var. tibetica*

Type of Variants	Count	Percent
DOWNSTREAM	36,274	7.39 %
EXON	63,080	12.86 %
nonsynonymous	34,536	7.04%
synonymous	26,333	5.37 %
stop gain	2,204	0.45%
stop loss	91	0.02 %
EXON;SPLICING	84	0.02 %
INTERGENIC	238,007	48.54 %
INTRON	71,298	14.54 %
SPLICING	828	0.17%
UPSTREAM	43,064	8.78 %
UPSTREAM;DOWNSTREAM	5,047	1.03%
UTR_3	19,193	3.91 %
UTR_5	13,424	2.74 %
UTR_5;UTR3	64	0.01%

Supplementary Table 4 Comparison of the CV error among different datasets used for population structure inferring in Admixture software

Datasets	Number of pruned SNPs	Cross-Validation (CV) errors				
		K = 1	K = 2	K = 3	K = 4	K = 5
5 Kbp	53,011	0.58412	0.54419	0.54256	0.54258	0.54727
10 Kbp	31,793	0.58100	0.54168	0.54016	0.54017	0.54479
15 Kbp	22,712	0.57935	0.54059	0.53909	0.53914	0.54439
20 Kbp	17,619	0.57766	0.53911	0.53761	0.53769	0.54220
25 Kbp	14,380	0.57545	0.53771	0.53623	0.53625	0.54121

Supplementary Table 5 Pairwise population fixation index (F_{ST}) among Tibetan poplar population along altitude gradient

	LL	DJ	PL	TM
LL		0.011	0.011	0.011
DJ	0.011		0.058	0.058
PL	0.066	0.058		0.006
TM	0.083	0.077	0.006	

Supplementary Table 6. Candidate environment associated genes identified by Bayenv2

GeneID	CHR	STR	END	Description	Environment factor
7495799	1	33107945	33111664	ACT domain-containing protein ACR6	Alt
18109687	1	40300229	40304163	BES1/BZR1 homolog protein 4	Srad
7480688	2	3287986	3291048	TP53-regulating kinase	Srad
7467222	2	8145999	8162107	E3 ubiquitin-protein ligase UPL1	Srad
7467223	2	8165095	8171769	kinesin-like protein KIN-14R	Srad
18096893	3	11100965	11105020	light-harvesting complex-like protein 3 isotype 1, chloroplastic	Srad
18098834	5	2843136	2868442	branched-chain-amino-acid aminotransferase-like protein 1	Alt, Tavg
18109775	5	2993094	2994220	histone H2AX	Srad
18098903	5	3401376	3411570	acyltransferase-like protein At1g54570, chloroplastic	Srad
7468855	5	8078885	8085745	phospholipase D delta	Alt
7476882	5	23036317	23042570	NADH dehydrogenase [ubiquinone] flavoprotein 2, mitochondrial	Srad
7493989	5	24881581	24887529	la-related protein 1B	Srad
7493996	5	24948208	24950371	uncharacterized LOC7493996	Srad
7485621	5	24989984	24991326	thioredoxin-like protein CDSP32, chloroplastic	Srad
7485640	5	25378645	25391849	NPC intracellular cholesterol transporter 1	Srad
7462243	6	23682528	23688009	geranylgeranyl transferase type-2 subunit alpha 1	Alt
7462291	6	24564943	24569258	uncharacterized LOC7462291	Srad
18100694	6	26214002	26218369	protein PHOX1	Srad
7468658	6	26222031	26224755	MADS-box transcription factor 47	Srad
7468660	6	26248280	26252031	hydroxyproline O-arabinosyltransferase 1	Srad
7468669	6	26349371	26353166	probable receptor-like protein kinase At1g80640	Srad
7489148	6	26603544	26606487	stromal cell-derived factor 2-like protein	Srad
7468690	6	26748943	26753022	transmembrane 9 superfamily member 8	Srad
112327926	6	26758471	26762881	uncharacterized LOC112327926	Srad
7468692	6	26768302	26775056	probable protein phosphatase 2C 60	Srad
7489161	6	26807445	26814907	protein RRC1	Srad
7471349	6	26885203	26890145	uncharacterized LOC7471349	Srad

7466733	7	13063411	13066816	5' exonuclease Apollo	Srad
7485034	7	13600298	13603975	beta carbonic anhydrase 5, chloroplastic	Alt
7462084	8	4404286	4407620	trihelix transcription factor ASIL2	Srad
7472447	8	4550352	4552894	protein trichome birefringence-like 33	Alt
7462100	8	4585873	4588801	laccase-12	Alt
18101560	8	10473404	10475728	protein MARD1	Alt
18102140	9	9871408	9873671	uncharacterized LOC18102140	Srad
7474953	9	11384837	11391549	protein DETOXIFICATION 45, chloroplastic	Srad
7475934	10	3988640	3994585	AP-5 complex subunit mu	Alt
7475555	10	13568705	13569214	E3 ubiquitin-protein ligase RHA2A	Srad
7459809	10	13576710	13582051	alpha-galactosidase	Srad
7468115	10	15791119	15792878	F-box protein At5g49610	Alt
7482208	12	14269875	14274717	proteasome subunit alpha type-2-A	Alt
7457781	15	10867474	10871602	pachytene checkpoint protein 2 homolog	Alt
18105959	15	13543391	13546165	squalene monooxygenase	Alt
112324382	15	13566621	13571204	uncharacterized LOC112324382	Alt
7481796	15	13574767	13581291	uncharacterized LOC7481796	Alt
18105953	15	13653212	13680221	chaperonin 60 subunit alpha 2, chloroplastic	Srad
7481806	15	13858532	13863900	coatomer subunit delta	Alt
112324539	16	1698259	1699239	putative receptor-like protein kinase At3g47110	Alt, Srad
18106754	17	581475	676167	rust resistance kinase Lr10	Srad
18110385	17	12287975	12290960	TMV resistance protein N	Tavg
18107455	17	12368649	12372727	disease resistance protein RPM1	Tavg
112324720	17	12375669	12378901	uncharacterized LOC112324720	Alt
7458750	18	10090971	10097480	probable serine incorporator	Srad

Table of Contents:

**Supplementary Fig.1**	**Page 7**
**Supplementary Fig.2**	**Page 8**
**Supplementary Fig.3**	**Page 9**
**Supplementary Fig.4**	**Page 10**
**Supplementary Fig.5**	**Page 11**

Supplementary Figure legends:

Supplementary Figure1: Pair-wise kinship matrix among 400 individuals. A set of 348 individuals were retained for subsequent analysis.

Supplementary Figure 2: The maximum likelihood trees reconstructed using Admixture software for the four sample sites.

Supplementary Figure 3: Comparison of nucleotide diversity of SNPs among different genomic regions. Four sample sites were indicated by four colors (Red: LL; Orange: DJ; Blue: PL; Green: TM).

Supplementary Figure 4: The LD decay pattern for four sample sites. Grey points represented 90% data.

Supplementary Figure 5: Annotation for genes associated with different environment variables. (a) Venn diagram for SNPs associated with altitude (blue), solar radiation (red) and average temperature (green). (b) Venn diagram for genes associated with different environment variables. (c) GO plot for all genes contained eaSNPs associated with environmental factors listed in Supplementary Table 6.

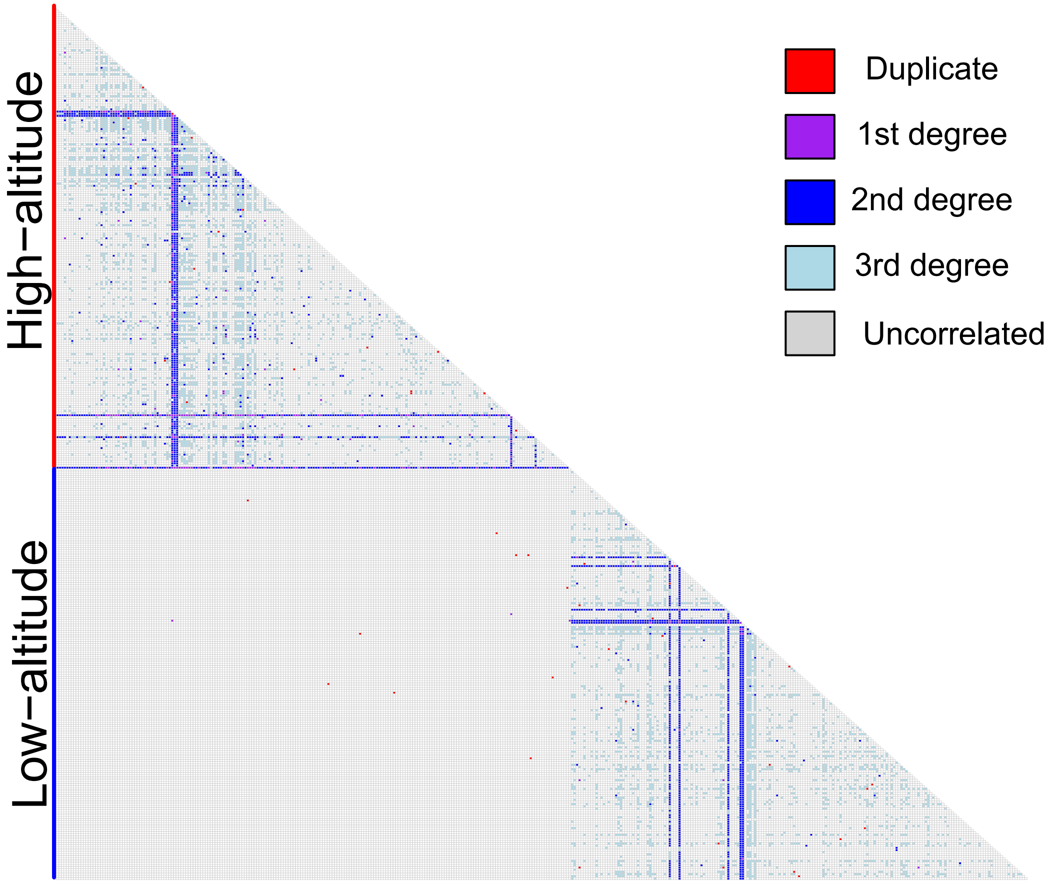
Duplicate
1st degree
2nd degree
3rd degree
Uncorrelated
High−altitude
Low−altitude

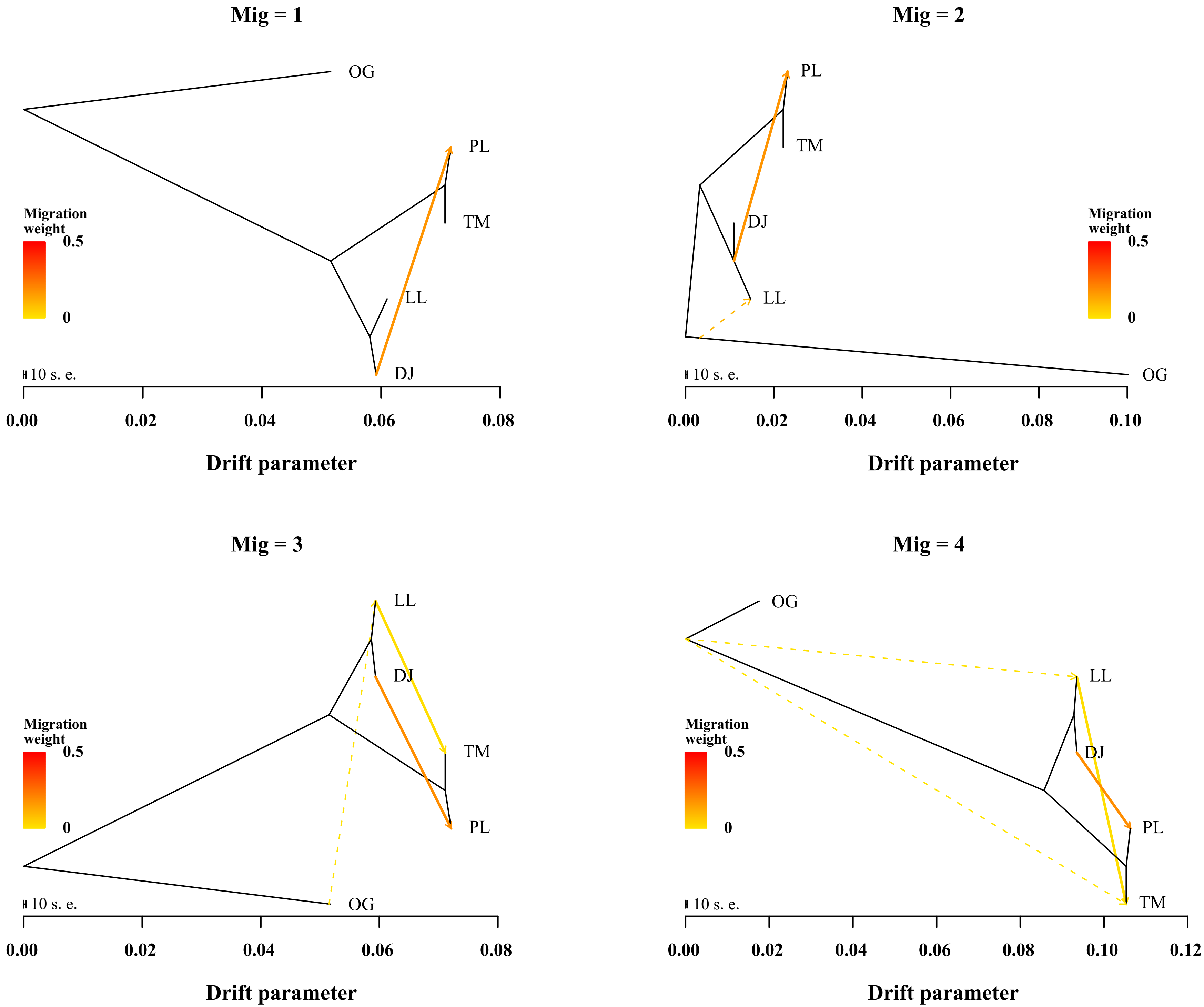
Mig = 1
OG
PL
TM
LL
DJ
Migration weight
0.5
0
10 s. e.
0.00
0.02
0.04
0.06
0.08
Drift parameter
Mig = 2
PL
TM
DJ
LL
OG
Migration weight
0.5
0
10 s. e.
0.00
0.02
0.04
0.06
0.08
0.10
Drift parameter
Mig = 3
LL
DJ
TM
PL
OG
Migration weight
0.5
0
10 s. e.
0.00
0.02
0.04
0.06
0.08
Drift parameter
Mig = 4
OG
LL
DJ
PL
TM
Migration weight
0.5
0
10 s. e.
0.00
0.02
0.04
0.06
0.08
0.10
0.12
Drift parameter

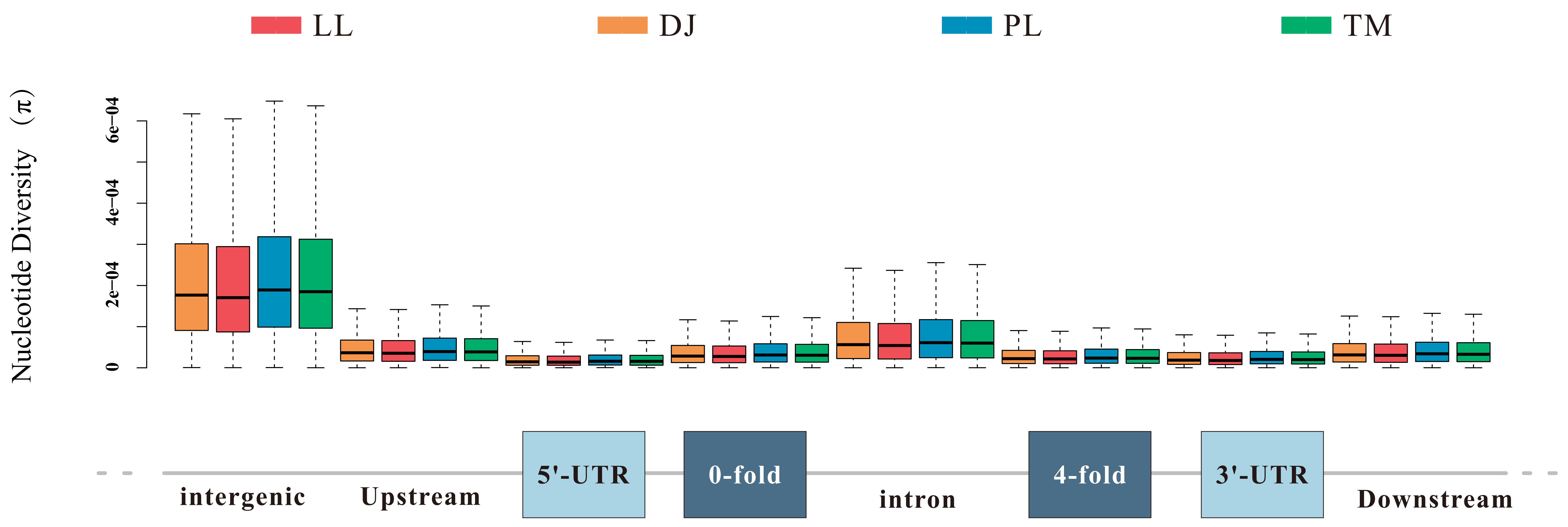

LL
DJ
PL
TM
Nucleotide Diversity (π)
0
2e−04
4e−04
6e−04
5'-UTR
0-fold
4-fold
3'-UTR
intergenic
Upstream
intron
Downstream

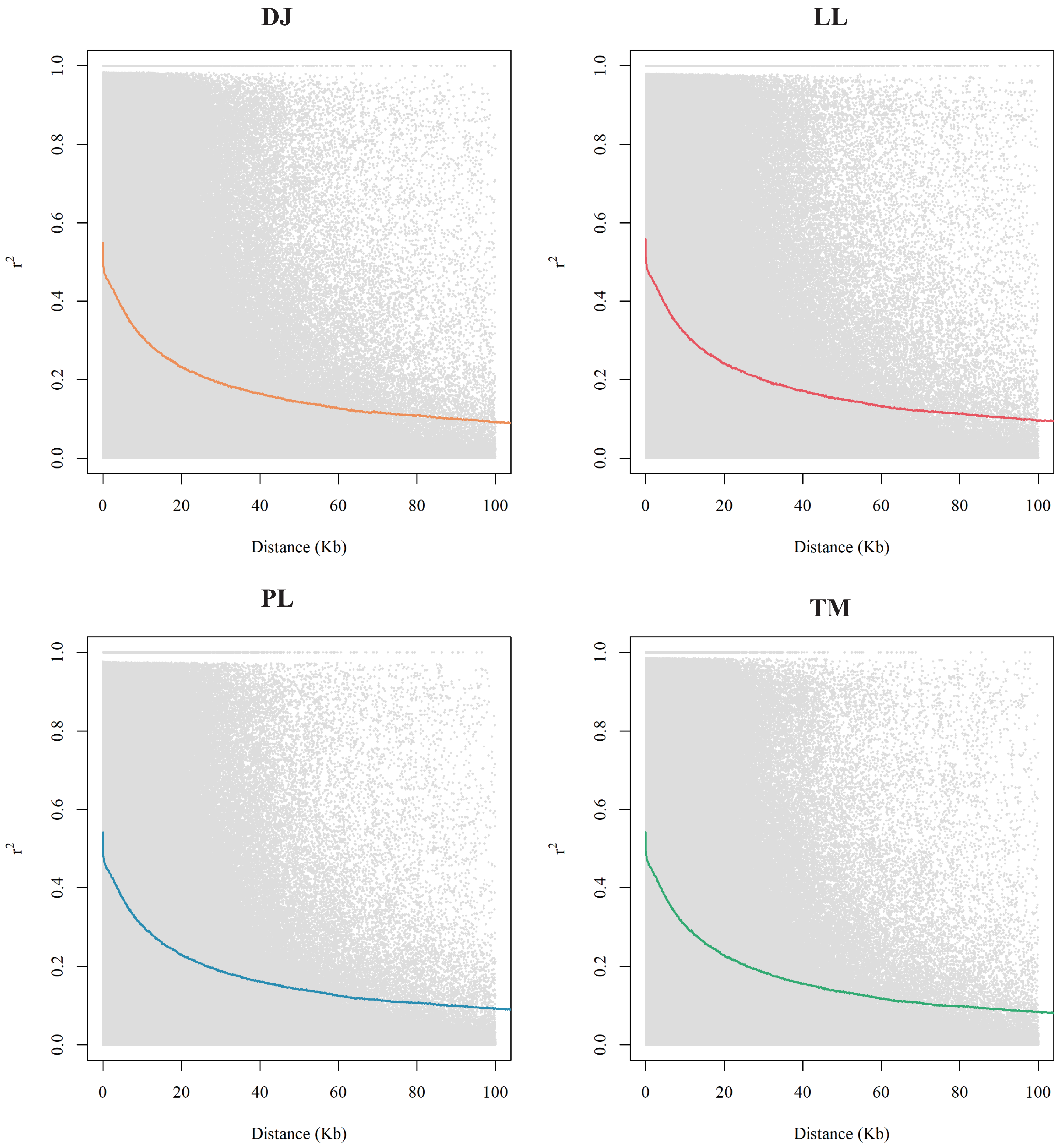

DJ
LL
PL
TM
r2
1.0
0.8
0.6
0.4
0.2
0.0
0
20
40
60
80
100
Distance (Kb)

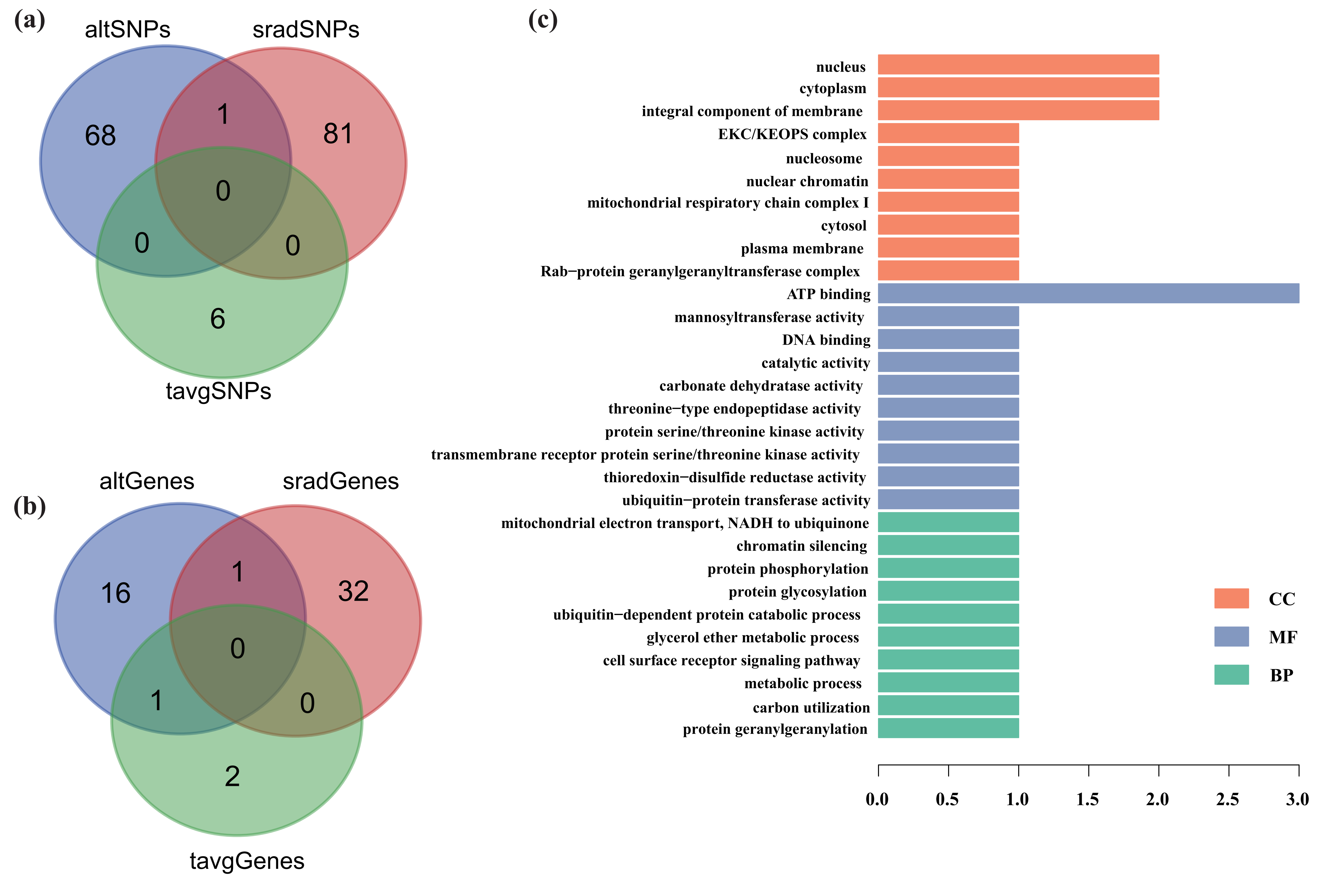

(a)
altSNPs
sradSNPs
tavgSNPs
68
1
81
0
0
0
6
(b)
altGenes
sradGenes
tavgGenes
16
1
32
0
1
0
2
(c)
nucleus
cytoplasm
integral component of membrane
EKC/KEOPS complex
nucleosome
nuclear chromatin
mitochondrial respiratory chain complex I
cytosol
plasma membrane
Rab−protein geranylgeranyltransferase complex
ATP binding
mannosyltransferase activity
DNA binding
catalytic activity
carbonate dehydratase activity
threonine−type endopeptidase activity
protein serine/threonine kinase activity
transmembrane receptor protein serine/threonine kinase activity
thioredoxin−disulfide reductase activity
ubiquitin−protein transferase activity
mitochondrial electron transport, NADH to ubiquinone
chromatin silencing
protein phosphorylation
protein glycosylation
ubiquitin−dependent protein catabolic process
glycerol ether metabolic process
cell surface receptor signaling pathway
metabolic process
carbon utilization
protein geranylgeranylation
0.0
0.5
1.0
1.5
2.0
2.5
3.0
CC
MF
BP
